# Effectiveness of Storytelling in Agricultural Marketing: Scale Development and Model Evaluation

**DOI:** 10.3389/fpsyg.2019.00452

**Published:** 2019-03-13

**Authors:** Hsiu-Ping Yueh, Yi-Lun Zheng

**Affiliations:** ^1^Department of Psychology, National Taiwan University, Taipei, Taiwan; ^2^Department of Bio-Industry Communication and Development, National Taiwan University, Taipei, Taiwan

**Keywords:** storytelling in marketing, marketing effectiveness, agricultural marketing, narrative processing, affect, measurement

## Abstract

Storytelling is a mode of communication in human interaction and is pervasive in everyday life. Storytelling in marketing is also a managerial application as a marketing strategy. Researchers of consumer psychology and marketing have devoted great efforts to developing theories and conducting empirical studies on this approach. However, in addition to narrative theories, many researchers are mainly concerned about the effect of telling a good brand story and its applications, such as advertising design and presentation. However, for those products that usually lacks branding, such as agricultural products, knowledge remains scarce about the relative impact of storytelling in marketing. Few researchers have explicitly developed a valid tool for measuring the effect of storytelling in marketing. To aid storytelling research in consumer psychology, this article conceptualized a construct of the effectiveness of storytelling in agricultural marketing and developed a measure with further validation. This scale consisted of 13 items with four subscales: narrative processing, affect, brand attitude, and purchase intention. The findings of this study supported a structural model with strong order among the four dimensions and good model fit. A discussion of the results and the theoretical and practical implications for consumer psychology and marketing practice are also addressed.

## Introduction

Economic development and changes in the production structure have led to changes in consumption patterns. For example, the marketing concept has changed from the production-orientation and selling-orientation of the early stage to marketing orientation as driven by consumer demand. In the past, product quality was regarded as the basic need of consumers. Enterprises were to pay attention to production while considering the needs of consumers and creating value to meet the requirements of consumers. The American Marketing Association (AMA) redefined marketing in 2004 as “an organizational function and a set of processes for creating, communicating, and delivering value to customers and for managing customer relationships in ways that benefit the organization and its stakeholders.” Compared to the definition developed in 1960, the new concept places greater emphasis on the importance of consumer-perceived value and customer relationship management because the transformation of consumption patterns and advancements in science and technology provide people with a wide range of information and all kinds of sensory stimulation. Gao (2008) pointed out that human beings are perceptive creatures. A good story with a strong appeal can stimulate the five senses. For example, listening to a story in the shopping environment can cause changes in consumption behavior (Hultén, 2015). Today, consumers no longer focus on the product alone, instead expecting an experience that can trigger and enrich their senses. In addition, sensory experience creates consumption value. As a result, storytelling has become a major trend in marketing in recent years.

Storytelling has been studied in psychology (e.g., McLean et al., 2007), sociology (e.g., Polletta et al., 2011), and organizational development (e.g., Morgan and Dennehy, 1997). Storytelling is an effective means of internal and external communication in the work field to transfer organizational culture and leadership styles to attract the attention of consumers and promote participation (Collison and Mackenzie, 1999). For internal management of an enterprise, managers communicate abstract business values through stories. In addition, the situation in a story is described to help employees understand ways to maintain the value of the enterprise (Fog et al., 2010). For external communication, a story engages the target audience. It can inspire consumers and enhance their loyalty (Pham et al., 2012). In the field of marketing, related research discusses persuasion in narrative advertising. For example, a narrative advertisement is one presented in the form of a story; this presentation has a different effect from other types of advertisements (Escalas, 2007; Polyorat et al., 2007). In consumer research, studies are mostly conducted to explore narrative ways in which consumers can express their consumption experiences (Shankar et al., 2001). In other words, consumers relate to the content with self-experiences after receiving the external information and create their self-identities (Polkinghorne, 1991). In brand development, a brand contains cultural meaning and the functions of self and group identity (Hirschman, 2010). Enterprises build brands through the narrative principle. Storytelling aims to deliver brand value. Brand stories of enterprises help in strengthening positive brand association (Lundqvist et al., 2013). Therefore, the power of a story should not be underestimated.

Agriculture is an industry with a long history. The term “marketing” was derived from farm product distribution (Weitz and Wensley, 2002). However, with changes in the structure of the agricultural industry and the concept of consumption, marketing no longer focuses on traditional marketing but on personal consumption and the social atmosphere of choice. On one hand, the market structure has been changed into an end-consumer orientation (Leroux et al., 2001), and agricultural marketing practices have become diverse. On the other hand, food safety crises continually emerge. Consumer awareness of food safety is so high that more and more consumers prefer to consume local food or organic products. The place of origin of agricultural products may imply authenticity, safety, health, and even nostalgia. Therefore, marketing can establish a relationship with consumers through the combination of signs and allusions related to the place of origin to strengthen brand attitude and purchase intention (Luceri et al., 2016). Moreover, limited by perishability and influenced by seasons and other features of agricultural products, significant breakthroughs in technology are difficult to achieve. However, aspects from the emotional side should be differentiated to solve the high isomorphism of agricultural products (Barrena and Sanchez, 2009). The deficiency problem, meaning that functional aspects cannot satisfy consumers, the use of emotion marketing should be able to solve it. In addition, a story has the power to impress people (McKee and Fryer, 2003).

Storytelling is now among the most popular marketing methods in the industry. In recent years, agricultural enterprises and small-scale farmers have been using storytelling in agricultural marketing to promote agricultural products. In agricultural marketing, storytelling is a common tool used on the internet and other digital marketing environments. However, previous studies on the application of stories in agricultural marketing are still limited, and most of that research lacks mechanisms for effectively measuring marketing effectiveness. Therefore, the purposes of this study were to develop an effectiveness scale for storytelling in agricultural marketing and to explore its factor structure. In addition to contributing to theory, the results of this study are expected to provide enterprises and farmers with effective guidelines for evaluating the effectiveness of storytelling in marketing in practice.

The research objectives of this study were as follows:

1. To develop a reliable and valid scale that captures the effectiveness of storytelling in agricultural marketing.
2. To apply this scale that measures consumers' attitudes toward and perceptions of storytelling in agricultural marketing.
3. To explore the four factors structural model (narrative processing, affect, brand attitude, and purchase intention) of and to test the effectiveness of the storytelling in agricultural marketing scale.

## Literature Review

### Storytelling and Consumer Behavior Model

Marketing is a process wherein individuals or organizations exchange products and value through production to obtain the goods or services they need and want (Kotler, 2000). From the perspectives of the seller and the consumer, marketing enables consumers to obtain commercial goods, services, and value. However, for marketers, the primary purpose of marketing is to drive consumers to buy products and thereby to make a profit. Therefore, the core concept of marketing management is to generate profit (Dickson, 1994).

Storytelling in marketing adopts marketing communication in the form of storytelling. The relationship between a story and consumer behavior should be further explained to specify the meaning of storytelling in marketing. The EKB model developed by Engel et al. (1978) proposed that consumers' buying behavior is a continuous activity consisting of information input, information processing, a decision-making process, variables affecting the decision-making process, and external influence. A story can significantly influence the information processing stage, which covers exposure, attention, comprehension, acceptance, retention, and memory. Chatman (1980) believed that a narrative is the basic mode of an organizational schema represented in the forms of language, paintings, dance movements, and music. In particular, multimedia presentations allow the easy exposition of information. Vivid stories enable consumers to imagine events and brand images in their minds. On one hand, a story can attract consumers' attention to the information; on the other hand, a conflict in a story can cause tension to enhance the reader's involvement (Fog et al., 2010). Able to influence people's emotions (Zemke, 1990), a story can also affect people's moods as a psychological factor (motivation) that determines whether the input information can be noticed. Emotional excitement makes people feel more focused.

Human culture and language learning enable people naturally to think and extract meaning from narratives. And narrative theory can inform the development of proposition of storytelling behaviors (Woodside, 2010). A common human experience constitutes a story prototype that has similar characteristics in a group (Loebbert, 2003). The classic character archetype delivers an original pattern of behaviors or personality traits. Some archetypes significantly influence the mode of people's thought in understanding the world (Polkinghorne, 1988). Therefore, a story becomes a tool for grasping the significance (Bruner, 1990). Some studies related to consumer storytelling show that in information processing, if brand or product information is included in the consumer story, it can be subconsciously accepted by people as moderate fun (Woodside et al., 2008). Therefore, having the power to persuade, a story is easily accepted (Loebbert, 2003) and can facilitate knowledge storage and retrieval. People store organized social knowledge with a narrative structure in memory (Schank and Abelson, 1995). A story can arouse the audience's emotions. As McGaugh (2003) pointed out, whether consciously or unconsciously, we often review experiences that have aroused our feelings.

People's affective response to advertising affects their attitudes toward the advertising and consequently their attitudes toward brands (Batra and Ray, 1986). Although affect can change peoples' brand attitudes, if consumers are not well informed of production information, then no credible reason is available to drive consumers to buy and change their original beliefs. Thus, the emotional effect cannot change their brand attitudes (Chandy et al., 2001). Therefore, in the case of sufficient information, a story can change consumers' emotions and influence their attitudes toward a brand, and this change can increase their purchase intentions and behaviors.

**Figure 1:**
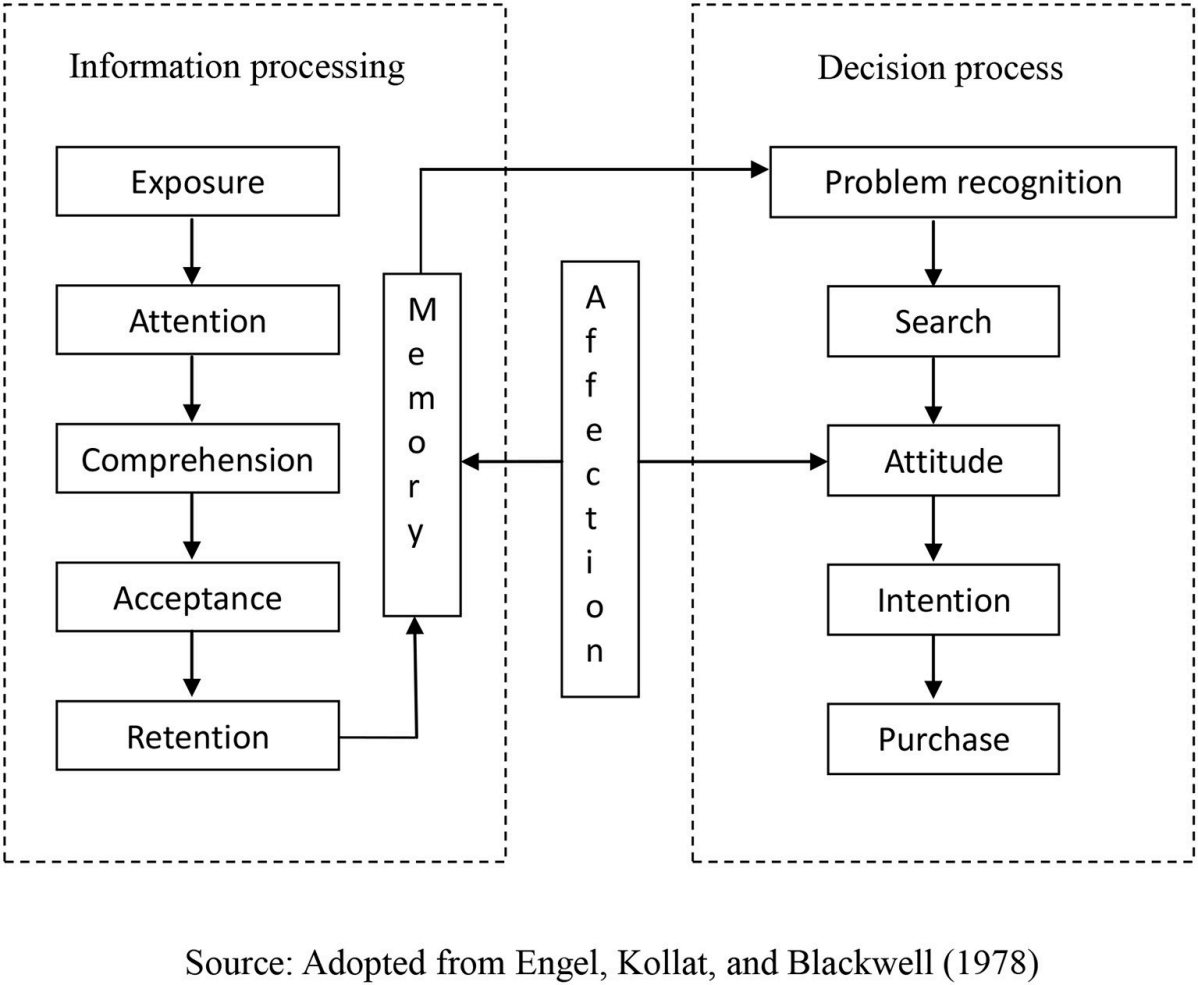
A modified model of story stimuli on consumer behavior.

This modified model regards a story as a kind of stimulus (see Figure 1), and the effect of storytelling in marketing is demonstrated as consumers' purchase decisions. Consumers' responses to story information constitute the main way to assess the benefits of storytelling in marketing. In marketing stimulus response, consumers experience three stages: cognitive, affective, and behavioral experiences (Kotler, 2003). In purchase decision making, consumers first process the information, and that processing triggers emotional responses. Consumers' attitudes toward advertising will then be directly transformed into attitudes toward the product in the advertisement (Brown and Stayman, 1992). In addition, their attitudes toward products or brands continue to affect their purchase intention. Storytelling in marketing regards a story (narrative) as a tool for communication that is different from the stimulus of the information form. Therefore, in this study, narrative processing is used as a substitute for information processing, and narrative processing, affect, brand attitude, and purchase intention are used as the measurement dimensions of the effectiveness of storytelling in marketing, as further discussed below.

#### Narrative Processing

Narrative processing is an important process for individuals in making consumption-related decisions. The EKB model is based on the information-processing model proposed by William McGuire (1978), which is divided into five steps. Information processing refers to the process of receiving a stimulus, interpreting, and storing it in memory. In other words, after individuals perceive external stimuli, they form memories through exposure, attention, comprehension, acceptance, and retention, leading to an action such as a purchase.

Storytelling is the most powerful means of communication. The link between story and information processing can be viewed through the abovementioned five steps when consumers receive and process the information. Storytelling in marketing delivers value and emotion through a story. It is a method of communication, and the public will understand stimulus inputs in story form (Fog et al., 2010). In the narrative process, stories about brands or product information can bring pleasure to consumers and lead consumers to relate to one or many story prototypes to achieve a high degree of understanding and acceptance (Holt, 2003; Woodside et al., 2008). In addition, the context of a story is rich in information; thus, organizing information with a narrative structure can help consumers to remember the important information.

Regarding agricultural product marketing, research has pointed out that telling stories about wines and chateaus can attract consumers' attention and allow them to connect it to a brand of wine (Beverland, 2005). Therefore, agricultural stories can be regarded as a means for communication between farmers and consumers, which helps consumers to understand and remember the content delivered by farmers easily.

#### Affect

Affect is a specific construct involving emotions, moods, or attitude, among which emotion has the most significant intensity. Emotion is a dynamic process between organism and environment (Lazarus, 1982). Mood has a lower intensity and longer duration. According to Batra and Ray (1986), affective responses are the moods and feelings caused by advertising. In the three phases of communication effects, the affect phase includes the willingness to look for additional information, generate interest in product attributes, and favorably evaluate the product or brand (Guiltinan et al., 1997).

Mehta (2000) pointed out that after receiving advertising information, consumers' preference degree influences their attention to the advertising. Therefore, when consumers receive marketing stimuli, the stimuli are reflected in the attention process. If a story is regarded as a stimulus, then it can attract attention and provide a captivating emotional experience (Woodside et al., 2008) and even move people (Zemke, 1990). Escalas and Stern (2003) believed that emotional responses include understanding of others and merging with other's feelings. This study proposes that stimulation by a story triggers consumers' attention, generates interest, and moves them.

#### Brand Attitude

The American Marketing Association (2008) defines a brand as a “name, term, design, symbol, or any other feature that identifies one seller's good or service as distinct from those of other sellers.” It is used to distinguish the differences in products or services provided by a certain manufacturer from those of a competitor. Swait et al. (1993) believed that brand marketing helps to communicate the attributes that contribute to the brand positioning and strengthen the reliability of brand positioning. In the case of information asymmetry, or advertising that lacks reliable sources of information, the brand name can reduce the cost of information and perceived risk such that consumers can identify the manufacturer that can provide the best product based on their experience, leading to repurchases (Hellier et al., 2003). A brand is not merely a name; it also implies a kind of value promise and transfers a kind of experience. The ultimate purpose of a brand is to construct a unique and strong emotional connection with consumers and increase consumer loyalty such that it becomes popular and has a good image (Kotler, 2012). The establishment of a brand helps consumers identify the enterprise and highlight the differences among product characteristics and the positioning of enterprises in the market. In a highly competitive market, considering that the heterogeneity of agricultural products is not extensive, when the differences in products are minor, consumers can make purchase decisions through the perception of the brand. Therefore, brand attitude can be used as an indicator for measuring marketing effectiveness.

Storytelling is an effective method of communicating brand value to consumers. In terms of explaining the value of a brand, traditional marketing methods and storytelling are not comparable (Lundqvist et al., 2013). Stories can help in transferring the brand value. Fog et al. (2010) pointed out that a story can make the core value of a brand easy to understand. Escalas (1998) stated that a story can attract and entertain consumers. A more important point is that a story can be used to communicate, display, and further persuade consumers to choose, buy, and use these products. A story can inform consumers of the brand and drive consumers to identify with its spirit and value. According to Fishbein and Ajzen (1975), brand attitude refers to consumers' preference or aversion to a particular brand. Howard and Sheth (1969) explained brand attitude by using brand preference, based on consumers' evaluations and trust in a brand. Therefore, this study suggests that a story can be used to inform consumers about a brand, transfer brand value, drive them to identify with the brand value, and trust in a brand.

#### Purchase Intention

Belch and Belch (2009) indicated that consumers' purchase intention is influenced by their purchase motivation, which is the extent to which consumers want to buy a product after considering brand attributes and features. Therefore, purchase intention refers to the probability that consumers are willing to make a purchase. Dodds et al. (1991) extended the conceptual model of Monroe and Krishnan (1985) and Zeithaml (1988), believing that purchase intention is affected by perceived quality and value. If consumers have a high perception of the value of a product, the purchase intention will increase. For example, in agricultural product consumption, if consumers hold a positive attitude toward organic food and want to gain benefits from it, then they will have a higher purchase intention (Teng and Lu, 2016). Consumers could show positive purchase intention if they have a higher commitment and desire to maintain valued relationships (Moorman et al., 1992). Therefore, consumers' purchase probability in the future will be relatively high (Garbarino and Johnson, 1999). In this study, purchase intention is defined as the probability and willingness of consumers to buy the product mentioned in the story after they are stimulated by the story.

### Narrative Persuasion

Storytelling pervades human life. People learn to communicate with others through body movements in a narrative form and co-create meaning after birth (Delafield-Butt and Trevarthen, 2015). Also, people tend to organize information in the form of stories, and such information is stored in the brain. When the new information is presented in a structure resembling the narrative form, similar to an individual's life experience, it becomes easier for people to understand (Adval and Wyer, 1998). Therefore, due to its structural characteristics, a story can help people to think. Escalas (1998) proposed that the chronology and causality of a narrative structure are two main factors that constitute a story. Events occur in the time dimension, and a relationship exists among story elements. In other words, roles and events are deployed at the beginning, the middle, and the end of the story, and causality exists between events. From this sequence, the audience can deduce the evolution and development of the story (Woodside et al., 2008).

Green and Brock (2002) examined narrative persuasion via transportation, defined as “an immersion into a text” (p. 702). Transportation in a narrative refers to information processing caused by a narrative combined with attention, imagery, and feelings. This mechanism influences belief and the persuasion principle of a story. In narrative transportation, readers may become immersed or lost in the story. Thus, negative cognitive response is reduced, and criticism of the product or counterargument is decreased. Meanwhile, the positive affective response is increased to cause a change in attitude (Green and Brock, 2002). Narrative transportation makes readers perceive the realism of the story and escape from the real world to put them into the story plot described by the author, and project themselves into the roles in the story. They regard the role experience in the story according to their experience to construct their own stories by strengthening their attitudes and beliefs (Green and Brock, 2002; Green, 2004). Therefore, a story can attract consumers and resonate with them (Escalas and Stern, 2003) to strengthen the persuasion effect. The literature in the field of advertising and marketing suggests that the story format can generate stronger positive affect and emotional response than the informational format in terms of enhancing purchase intention (Adval and Wyer, 1998; Carlsson Hauff et al., 2014).

Agricultural stories usually involve plots wherein farmers stick to their convictions to adhere to their original intention of engaging in agricultural practice even in the face of difficult challenges. For example, agricultural stories published by the Agricultural Bureau of Kaohsiung tell of Mr. Chung, a dragon fruit farmer, who engaged in the cultivation of dragon fruit after he retired to take care of a child suffering from a rare disease. He lost his pension, but he still adhered to friendly and safe cultivation in order to care for his child. Finally, his business expanded to Canada, Dubai, and other countries. In this agricultural story, consumers can be deeply affected by the protagonist's situation because they may associate it with their own experiences in times of crisis and difficulty. In addition, sympathy and empathy responses influence their attitudes (Escalas and Stern, 2003). Green and Brock (2002) pointed out that under high narrative transportation, readers may have a greater affinity for a sympathetic protagonist. As shown in the case above, if consumers are deeply attracted to a story, then they may identify with a farmer caring for agricultural products in a way similar to how he takes care of his children, and even support him by purchasing the products.

According to transportation theory, when consumers receive narrative information, it may affect their attitudes and behavioral intentions; consequently, consumers will show more story-consistent beliefs. Escalas (2007) believed that mental simulation is usually presented in the form of a narrative. In a hypothetical case, by combining re-experience, reoccurrence, and imagining of previous and future events, the audience experiences the story as a simulation running in the mind. Mental simulation affects narrative transportation to influence the affection (Escalas, 2004a). Fiske (1982) suggested that positive affect increases when the received information matches the schema. Deighton et al. (1989) also pointed out that if the advertising is closer to the narrative structure, then the advertising plot arouses consumers' emotional responses easily. As the positive experience is transferred, narrative processing may arouse positive affection even more. However, a narrative has either a positive or negative effect on affection. Consumers are more strongly captivated by exciting, warm, and other positive experiences of stories, while they are not attracted to dull and boring stories (Escalas et al., 2004).

Narrative processing affects the emotions and influences the brand attitudes of consumers. The characteristics of the story structure form links to goals related to the self-concept, such as narrative processing and making inferences about the brand (Woodside et al., 2008). Therefore, a narrative message can cause a change of attitude more easily than can an information message (Grace and Kaufman, 2013). Studies related to brand narrative have also shown that stories help consumers to aquire the brand experience. After receiving narrative information, consumers attempt to relate the information to their memories. Narrative processing can strengthen the link between the self and the brand and create significance. When the link between the self and the brand is improved, high purchase intention may be generated (Escalas, 2004b). Related studies have noted that when consumers hold a positive attitude toward organic food, consumers' purchase intention of organic food is increased, too (Chen, 2007). Therefore, brand attitude also influences purchase intention.

Moreover, when emotion is evoked by narrative processing, it influences the consumers' brand attitude and the purchase intention (Yi, 1990). The positive emotion triggered by narrative transportation helps brand evaluation (Escalas, 2004a). Charters et al. (2009) showed that after consumers listened to stories about wineries and wines, their sympathy and interest were provoked and their loyalty to the brand improved. Escalas (2004a) also pointed out that when consumers see narrative advertisements, they imagine themselves possibly gaining benefits from the product through the generation of a mental image. They have a strongly positive emotion in narrative transportation, which increases the likelihood of buying the product. Therefore, the positive emotion of consumers toward agricultural products or brands affects their willingness to consume those products.

**Figure 2:**
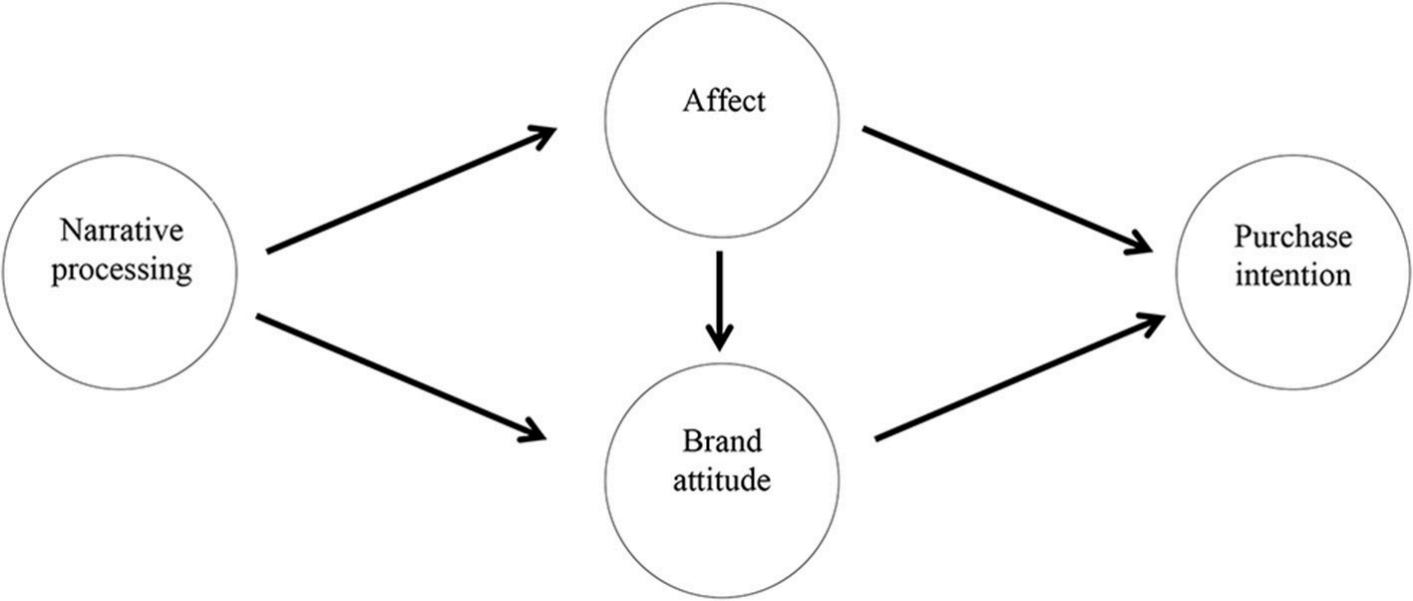
Conceptual model of effectiveness of storytelling in agricultural marketing dimensions.

Based on the review of the literature, this study concludes four effectiveness dimensions from the EKB model and narrative function: narrative processing, affect, brand attitude, and purchase intention. Relationships among the dimensions of this model are assumed to further verify the interaction among the dimensions (see Figure 2). The hypotheses for testing the model are proposed as follows:

H1: Narrative processing has a positive impact on affect.
H2: Narrative processing has a positive impact on brand attitude.
H3: Affect has a positive impact on purchase intention.
H4: Affect has a positive impact on brand attitude.
H5: Brand attitude has a positive impact on purchase intention.
H6: Affect mediates the relationship between narrative processing and subordinate purchase intention.
H7: Affect mediates the relationship between narrative processing and subordinate brand attitude.
H8: Brand attitude mediates the relationship between narrative processing and subordinate purchase intention.
H9: Brand attitude mediates the relationship between affect and subordinate purchase intention.

## Method

This study attempted to develop a scale and to test the measure's factor structure and validity. In this study, key concepts related to storytelling in marketing, agricultural marketing, and marketing effectiveness from agricultural marketing-related literature were addressed and the items of this scale were generated. After item generation, three experts in the fields of agricultural marketing and communication were invited to establish the content and face validity of the scale. Confirmatory factor analysis (CFA) was used to examine the reliability and validity of the effectiveness of storytelling in agricultural marketing scale, the relationships among factors were analyzed with structural equation modeling (SEM), and the model fit was verified. We randomly select half of the sample (*N* = 190) to conduct the CFA and the other sample (*N* = 190) to test additional hypotheses.

### Subjects

Convenient sampling was used to recruit participants from people enrolled in the “Storytelling in Agricultural Marketing Workshop” held by the Council of Agriculture. Respondents of this study were fully informed about the research purpose; and they returned the questionnaires voluntarily. No additional ethical approval was required in this study, in accordance with national and institutional requirements. In total, 380 valid survey responses were collected. There were 161 man and 219 women. The participants were business owners and farmers (54), office workers and employees (39), students (271), and unemployed people (16), and the average age was 26 years (SD = 8.77).

### Measures

The “Storytelling in Agricultural Marketing Effectiveness Scale” (SAMES) developed by the researchers included sets of questions on four factors: narrative processing, affect, brand attitude, and purchase intention. It was administered to the participants in this study. This scale consisted of 13 items, to which participants responded on a six-point Likert-type scale (ranging from 1 = “strongly disagree” to 6 = “strongly agree”).

### Data Analysis

The collected survey data were analyzed in SPSS for Windows version 15.0, and structural equation modeling (SEM) was applied to test the proposed model using LISREL 8.70.

## Results and Disscusion

### Descriptive Statistics and Item Analysis

Appendix 1 presents results of the item analysis of the SAMES. The item mean (M) scores ranged from 4.66 to 5.18, with the highest-scoring item being “storytelling in agricultural marketing can make it easier for consumers to understand the content.” (M = 5.18), and the lowest being “storytelling in agricultural marketing can cause consumers to buy agricultural products” (M = 4.66). This might imply that consumers act after several careful evaluations, and that purchase is the final step in the consumer decision-making process. Therefore, driving consumers to buy is very difficult. The standard deviation (SD) of this scale ranged from 0.85 to 1.17. The absolute value of the skewness of the scale (−1.34–0.19) was smaller than 3, and the absolute value of kurtosis (−0.83–2.95) was smaller than 10. These findings indicated that the measured items were appropriate and that the data adhered to the assumption of normal distribution. To verify the item discrimination degree, the independent samples *t*-test was used to detect whether a difference existed between the high (top 27%) and low (last 27%) score groups. The analysis showed that each item in this study reached the significance level (α = 0.05). The findings indicated that all measured items had a proper discrimination index. To test item-total correlation, the Pearson correlation coefficients of individual items and the total score were obtained, and the results showed that the correlation coefficient index between the item and the total score was higher than the critical ratio of 0.5. The reliability coefficient (Cronbach's alpha) for the scale was 0.947, which showed good internal consistency (DeVellis, 2012).

### Exploratory Factor Analysis

Exploratory factor analysis (EFA), using principal axis factor analysis with promax rotation, was conducted to determine the appropriate factor structure for the scale. A four-factor solution that significantly met Bartlett's test of sphericity (χ^2^ = 1983.160,) and the Kaiser-Meyer-Olkin (KMO) measure of sampling adequacy (0.918) was determined for the scale. The factor structure had eigenvalues >1.0 and explained 80.442% of the variance. One item that had very low loading was removed from the final scale. The loadings of narrative processing ranged from 0.513 to 0.890, those of affect ranged from 0.627 to 0.929, those of brand attitude ranged from 0.531 to 0.966, and those of purchase intention ranged from 0.720 to 0.953, providing the optimal factor structure.

### Confirmatory Factor Analysis

As four latent factors in the SAMES were found by exploratory factor analysis, this study further examined the discriminant validity of the factors to ensure that the factor structures derived from EFA were accurate. Considering that the variables were ordinal data and that some of the kurtosis values were >1, it would not have been suitable to use the maximum-likelihood estimation (MLE), as suggested by Muthén and Kaplan (1985); (Muthen and Kaplan, 1992). Therefore, confirmatory factor analysis (CFA) with Sattora-Bentler scaled chi-square (DiStefano, 2002) was used to test the factorial validity.

Preliminary fit criteria have two items in the standard test, namely, the testing error variance and the standardized solution. Results showed that no negative error variation existed, and the correlative value between the two parameters was neither >1 nor <-1 (see Appendix 2). In the structural model, parameters did not go beyond the acceptable scope; that is, no offending estimate was available. Therefore, the scale in this study conforms to the preliminary fit criteria.

Results showed that the fit of the overall model and observed data had a Satorra-Bentler chi-square value of 131.75 at the significance level, yet the fit between the observed data and theoretical model was poor. However, in practice, with the effect of sample size, the index reached significance, as often happens, and it could be viewed merely as a reference. With the exception that χ^2^ reached the significance level, the model-fit indices for this scale revealed a good fit to the data: RMSEA = 0.081, CFI = 0.99, NFI = 0.97, NNFI = 0.98, and SRMR = 0.056(see Table 1).

**Table 1:** Model fit criteria summary table of the storytelling in agricultural marketing effectiveness scale.

	**Recommended criteria**	**References**	**Results**
χ2	The smaller the better		236.85
Satorra-Bentler χ2	The smaller the better		131.75
df			59
RMSEA	≤0.08	Hair et al., 2010	0.081
CFI	>0.90	Hair et al., 2010	0.99
NFI	≥0.95	Hu and Bentler, 1999	0.97
NNFI	>0.90	Bentler and Bonett, 1980	0.98
SRMR	≤0.08	Hu and Bentler, 1999	0.056

Furthermore, the confidence intervals of the paired correlations among latent variables ranged from 0.99 to 0.70, indicating that the four factors had discriminant validity. In addition, the average variance extracted from the scale ranged from 0.64 to 0.70, which is higher than the standard value of 0.5 (Fornell and Larcker, 1981; Bagozzi and Yi, 1988). This indicated that the latent variables were subject to higher contribution of the observed variables than the contribution of errors, and confirmed that the scale exhibited good convergent validity. In addition, the composite reliability (CR) of the four latent variables of narrative processing (0.85), affect (0.84), brand attitude (0.88), and purchase intention (0.87) also yielded good internal consistency estimates (see Table 2).

**Table 2:** Item reliability, composite reliability, and average variance extracted of the storytelling in agricultural marketing effectiveness scale.

**Dimensions**	**Items**	**Item reliability**	**Composite reliability**	**Average variance extracted**
Narrative processing	I believe that storytelling in agricultural marketing can promote communication between farmers and consumers.	0.64	0.85	0.65
	I believe that storytelling in agricultural marketing can make it easier for consumers to remember the content.	0.69		
	I believe that storytelling in agricultural marketing can make it easier for consumers to understand the content.	0.62		
Affect	I believe that storytelling in agricultural marketing can move consumers.	0.44	0.84	0.64
	I believe that storytelling in agricultural marketing can attract consumers' attention.	0.79		
	I believe that storytelling in agricultural marketing can interest consumers in agricultural products.	0.81		
Brand attitude	I believe that storytelling in agricultural marketing can improve the incomes of farmers.	0.58	0.88	0.66
	I believe that storytelling in agricultural marketing can transfer the brand value.	0.83		
	I believe that storytelling in agricultural marketing can make consumers to know brands.	0.53		
	I believe that storytelling in agricultural marketing can make consumers identity with the spirit and value of the brand.	0.71		
Purchase intention	I believe that storytelling in agricultural marketing can improve the purchase intention of consumers.	0.73	0.87	0.70
	I believe that storytelling in agricultural marketing can cause consumers to buy agricultural products.	0.71		
	I believe that storytelling in agricultural marketing can cause consumers to recommend agricultural products to their relatives and friends.	0.66		

### Structural Model

This study further adopted structural equation modeling (SEM) to test the theoretical dimensions of the scale and the model fit.

Results showed that the fit of the overall model and observed data had a Satorra-Bentler chi-square value of 151.16 at the significance level. The SEM model produced good fit indices, as presented in Table 3: RMSEA = 0.079, CFI = 0.98, NFI = 0.98, NNFI = 0.98, and SRMR = 0.047.

**Table 3:** Model fit criteria summary table of the storytelling in agricultural marketing effectiveness scale.

	**Recommended criteria**	**References**	**Results**
χ2	The smaller the better		233.47
Satorra-Bentler χ2	The smaller the better		151.16
df			60
RMSEA	≤0.08	Hair et al. (2010)	0.086
CFI	>0.90	Hair et al. (2010)	0.98
NFI	≥0.95	Hu and Bentler (1999)	0.98
NNFI	>0.90	Bentler and Bonett (1980)	0.97
SRMR	≤0.08	Hu and Bentler (1999)	0.044

**Figure 3:**
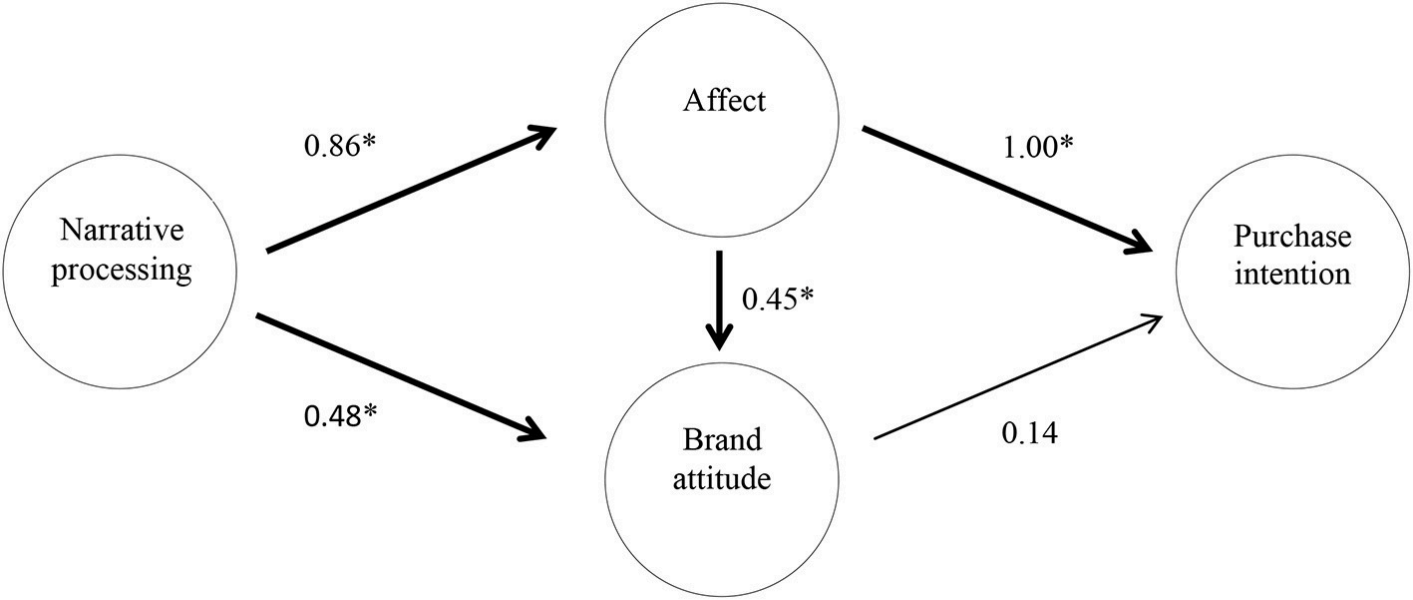
Conceptual model of effectiveness of storytelling in agricultural marketing dimensions. Variables that tested significant appear in bold (^*^p < 0.05).

The hypotheses that the research model established were tested and the relationships among narrative processing, affect, brand attitude, and purchase intention were analyzed. Figure 3 presents the path coefficients of the theoretical structure model. The results showed that only brand attitude did not predict purchase intention; H5, H8, and H9 were thus rejected (see Table 4). For the other direct effects, as revealed in Figure 3, narrative processing had a positive impact on brand attitude; affect had a positive impact on brand attitude; narrative processing had a positive impact on affect; and affect had a positive impact on purchase intention. Therefore, H1 to H4 were all supported. Furthermore, narrative processing indirectly predicted purchase intension and brand attitude, respectively, through affect; H6 was thus supported with a full mediation effect (0.70^*^), and H7 was supported with a partial mediation effect (0.25^*^).

**Table 4:** Summary of results of hypothesis testing.

**Hypothesis**	**Results**
H1: Narrative processing → Affect	Supported
H2: Narrative processing → Brand attitude	Supported
H3: Affect → Brand attitude	Supported
H4: Affect → Purchase intention	Supported
H5: Brand attitude → Purchase intention	Rejected
H6: Narrative processing → Affect → Purchase intention	Supported
H7: Narrative processing → Affect → Brand attitude	Supported
H8: Narrative processing → Brand attitude → Purchase intention	Rejected
H9: Affect → Brand attitude → Purchase intention	Rejected

## Conclusions

This study aimed to develop the “Storytelling in Agricultural Marketing Effectiveness Scale” (SAMES) and explore the structural model of effectiveness. This scale consisted of 13 items with four subscales: narrative processing, affect, brand attitude, and purchase intention. The structural model was tested and confirmed to have satisfactory model fit. The findings of this study have theoretical and practical implications, which are discussed as follows.

### Implications for Practice

First, this study developed the SAMES, which had good reliability and validity. Results showed this scale to have four latent variables: narrative processing, affect, brand attitude, and purchase intention. The scale achieved an excellent level of coefficients in the CFA in various tests. Generally, having good consistency reliability, factor structure, and construct validity, this scale can be used as a tool for evaluating the success of storytelling in marketing and as a reference for farmers or agricultural marketers to evaluate their marketing effectiveness.

Second, this study also explored the relationships among the variables in the effectiveness of storytelling in marketing. Through further construction and verification of the theoretical model of the SAMES, results showed that brand attitude did not significantly affect purchase intention. Unlike in other industries, the concepts of brand building and enterprise development are still emerging in the agricultural industry. On agricultural electronic commerce websites, agricultural stories do not focus on the brand information, emphasizing instead the role of the farmer and illustrating the farming practices. This approach is considerably different from other types of electronic commerce, such as those of 3C products, luxury goods, and food and drink, which always emphasize the brand and the product specifications. Moreover, since agriculture touches the lives of nearly everyone every day, people tend to seek agricultural products with relatively lower prices. Therefore, consumers tend not to evaluate the brand or other marketing factors while making purchase decisions. Although narratives can strengthen the linkage between brand and consumer (Escalas, 2004b), the characteristics of agricultural brands are not apparent; thus, this study found that storytelling cannot promote consumer awareness of brand information and affect their purchase intentions.

Third, the ultimate goal of marketing is to persuade consumers to buy products or services. This study explored the pathways of factors that influence consumers' intention. Results showed that narrative processing indirectly affected purchase intention through consumers' emotional arousal responses. Affect plays a mediating role in agricultural marketing through storytelling. After being exposed to the agricultural stories, consumers will have emotional responses, such as projecting their experiences into the events experienced by the main character in the story, being moved by the story, and so on. Thus, storytelling can further drive consumers to buy agricultural products. This study showed that affect is an important element in the consumers' purchase decision-making process. This finding is consistent with those of previous studies, such as that of Barrena and Sanchez (2009), who argued that agricultural marketing must strengthen the linkage between affect and product. McKee and Fryer (2003) also pointed out that good stories could move people's hearts and excite people's emotions. The current study also contended that agricultural stories should focus on strengthening this linkage to improve marketing effectiveness. Managers should focus on the factors that can induce positive emotions to develop a good strategy for storytelling in agricultural marketing.

### Implications for Theory

This study revealed the relationships among the factors affecting the effectiveness of storytelling in agricultural marketing. Van Laer et al. (2014) reviewed 76 papers related to narrative persuasion published over the preceding 20 years and performed meta-analysis on 132 models related to narrative transportation. They also extended the transportation-imagery model proposed by Green and Brock (2002), constructed the extended transportation-imagery model (ETIM), and explored how the characteristics of the storytellers and story listeners affect the narrative transportation process and in turn influence the generation of cognition, affective response, belief, attitude, and intention. However, because of the low magnitudes of intercorrelations, Green and Brock's study could not further clarify the correlations among those factors to determine effectiveness. Filling this gap, the results of this current study strongly support the existence of a potential relationship among narrative perception, affect, attitude, and the path that predicts purchase intention effectiveness. This is similar to the hierarchy of effects in advertising; while watching advertising, consumers will analyze its message, further forming a particular affect, and then make purchase decisions through the assurance of the attitude to the advertisement and the product (Lavidge and Steiner, 1961). In concept, the strategy of storytelling in marketing is more novel than traditional advertising; both of them fall within the scope of marketing persuasion. The findings of this study support the existence of a strong order among the four dimensions, one that can be referenced for future study.

## Limitations and Future Research

Although this study developed a valid measure of the effectiveness of storytelling in agricultural marketing, the SAME, and although the final model fit the data well, the findings still should be considered in light of the limitations of the survey method. First, this study recruited participants in four workshops as the sample, which may call into question the external validity of the results. Also, the generalization of the results may be restricted. Based in part on these limitations, it is suggested that the proposed model be tested in future research considering broader perspectives. For example, future studies could investigate the main buyers of agricultural products in households and extend the variety of participants' traits, such as organic or traceable agricultural product consumer groups, to collect their opinions of and preferences for storytelling in marketing. Furthermore, future studies could further explore the effects of different types, components, and story appeals of agricultural stories on the marketing effectiveness based on the factors and structural model developed in this study. It is recommended that researchers further examine mediators other than affect in the current model. Nevertheless, the findings of this study provide a comprehensive understanding of how narrative processing, affect, and brand attitude work together to influence consumers' purchase intention of agricultural products. The study also contributes valuable insights into how farmers and all actors involved in the complex agricultural market use the storytelling strategy in agricultural marketing.

## Author Contributions

H-PY developed the concept and design of this study. Data was collected and analyzed by Y-LZ. All authors interpreted the results. Y-LZ drafted the manuscript under the supervision of H-PY, and H-PY provided critical revisions. All authors approved the final version of the manuscript for submission.

### Conflict of Interest Statement

The authors declare that the research was conducted in the absence of any commercial or financial relationships that could be construed as a potential conflict of interest.
