# Supplementary material for: Effectiveness of Storytelling in Agricultural Marketing: Scale Development and Model Evaluation
Source: Front Psychol. 2019 Mar 13;10:452. doi: 10.3389/fpsyg.2019.00452 (PMC6424896; doi:10.3389/fpsyg.2019.00452)
Supplement: Supplementary file 1 [file Table_1.docx]

**Appendix 1 Item Analysis Summary Table of the Storytelling in Agricultural Marketing Effectiveness Scale**

| Items | N | Min | Max | Mean | SD | Skewness | Kurtosis | t-value | r |
| --- | --- | --- | --- | --- | --- | --- | --- | --- | --- |
| I believe that storytelling in agricultural marketing can improve the incomes of farmers. | 190 | 1.0 | 6.0 | 4.83 | 0.97 | -0.808 | 0.914 | 14.84 | 0.68** |
| I believe that storytelling in agricultural marketing can transfer the brand value. | 190 | 1.0 | 6.0 | 5.15 | 0.91 | -1.159 | 1.717 | 15.89 | 0.83** |
| I believe that storytelling in agricultural marketing can promote communication between farmers and consumers. | 190 | 2.0 | 6.0 | 5.07 | 0.88 | -0.670 | -0.058 | 22.32 | 0.74** |
| I believe that storytelling in agricultural marketing can make it easier for consumers to remember the content | 190 | 1.0 | 6.0 | 5.16 | 0.88 | -1.129 | 1.944 | 15.50 | 0.76** |
| I believe that storytelling in agricultural marketing can make it easier for consumers to understand the content. | 190 | 1.0 | 6.0 | 5.18 | 0.85 | -1.146 | 2.309 | 14.24 | 0.72** |
| I believe that storytelling in agricultural marketing can make consumers know brands. | 190 | 1.0 | 6.0 | 5.08 | 0.95 | -1.344 | 2.948 | 14.65 | 0.68** |
| I believe that storytelling in agricultural marketing can make consumers identify with the spirit and value of the brand. | 190 | 1.0 | 6.0 | 5.03 | 0.97 | -0.872 | 0.603 | 16.42 | 0.81** |
| I believe that storytelling in agricultural marketing can move consumers. | 190 | 1.0 | 6.0 | 4.85 | 1.17 | -0.936 | 0.567 | 18.46 | 0.68** |
| I believe that agricultural story marketing can attract consumers’ attention. | 190 | 1.0 | 6.0 | 5.14 | 0.93 | -1.247 | 2.151 | 14.94 | 0.78** |
| I believe that storytelling in agricultural marketing can interest consumers in agricultural products. | 190 | 2.0 | 6.0 | 5.03 | 0.90 | -0.805 | 0.408 | 14.84 | 0.80** |
| I believe that storytelling in agricultural marketing can improve the purchase intention of consumers. | 190 | 2.0 | 6.0 | 4.95 | 0.94 | -0.678 | 0.182 | 18.00 | 0.75** |
| I believe that storytelling in agricultural marketing can cause consumers to buy agricultural products. | 190 | 2.0 | 6.0 | 4.66 | 1.02 | -0.193 | -0.832 | 20.69 | 0.70** |
| I believe that storytelling in agricultural marketing can cause consumers to recommend agricultural products to their relatives and friends. | 380 | 1.0 | 6.0 | 4.82 | 1.04 | -0.837 | 0.541 | 16.82 | 0.74** |

| **Appendix 2 Complete Standardized Solutions Summary Table of the Storytelling in Agricultural Marketing Effectiveness Scale** | | | | |
| --- | --- | --- | --- | --- |
| Dimensions/Items | Narrative  processing | Affect | Brand  attitude | Purchase  intention |
| I believe that storytelling in agricultural marketing can promote communication between farmers and consumers. | 0.80 |  |  |  |
| I believe that storytelling in agricultural marketing can make it easier for consumers to remember the content. | 0.83 |  |  |  |
| I believe that storytelling in agricultural marketing can make it easier for consumers to understand the content. | 0.79 |  |  |  |
| I believe that storytelling in agricultural marketing can move consumers. |  | 0.66 |  |  |
| I believe that storytelling in agricultural marketing can attract consumers’ attention. |  | 0.89 |  |  |
| I believe that storytelling in agricultural marketing can interest consumers in agricultural products. |  | 0.90 |  |  |
| I believe that storytelling in agricultural marketing can improve the incomes of farmers. |  |  | 0.76 |  |
| I believe that storytelling in agricultural marketing can transfer the brand value. |  |  | 0.91 |  |
| I believe that storytelling in agricultural marketing can make consumers know brands. |  |  | 0.72 |  |
| I believe that storytelling in agricultural marketing can make consumers identify with the spirit and value of the brand. |  |  | 0.84 |  |
| I believe that storytelling in agricultural marketing can improve the purchase intention of consumers. |  |  |  | 0.85 |
| I believe that storytelling in agricultural marketing can cause consumers to buy agricultural products. |  |  |  | 0.84 |
| I believe that storytelling in agricultural marketing can cause consumers to recommend agricultural products to their relatives and friends. |  |  |  | 0.81 |
